# Diagnostic Consideration for Sinonasal Wegener's Granulomatosis Clinically Mistaken for Carcinoma

**DOI:** 10.1155/2013/839451

**Published:** 2013-09-09

**Authors:** Cristina La Rosa, Carmela Emmanuele, Maria Grazia Tranchina, Massimo Ippolito, Sebastiano Cosentino, Vincenzo Saita, Giuseppina Improta, Filippo Fraggetta

**Affiliations:** ^1^Department of Pathology, AOE Canizzaro, Via Messina 829, 95126 Catania, Italy; ^2^Department of Nuclear Medicine, AOE Canizzaro, Via Messina 829, 95126 Catania, Italy; ^3^Department of Cervicofacial Surgery, AOE Canizzaro, Via Messina 829, 95126 Catania, Italy; ^4^Laboratory of Clinical Research and Molecula Diagnostics, IRCCS Centro di Riferimento Oncologico della Basilicata (CROB) Via Padre Pio 1, 85028 Rionero in Vulture, Potenza, Italy

## Abstract

We report a case of Wegener's granulomatosis clinically mistaken for carcinoma in a 21-year-old girl presenting with an ulcerated mass of the nasopharynx associated with enlarged laterocervical nodes. The lesion was clinically suspected as malignant on the basis of clinical and radiological findings (namely, computed tomography scan and positron emission tomography). However, multiple biopsies were not conclusive for malignancy showing histological change suggestive of Wegener's granulomatosis. A serum determination of cANCA supported the diagnosis of Wegener's granulomatosis. Clinical findings and image studies suggested an erroneous diagnosis of malignancy whereas a definitive diagnosis of Wegener's granulomatosis was achieved only after repeated biopsies thus leading to a correct therapeutic approach. The Wegener granulomatosis must be added to the list of the differential diagnoses of the masses of the nasopharynx associated with or without enlarged laterocervical nodes.

## 1. Introduction

Wegener's granulomatosis (WG) is a systemic, autoimmune disease histologically characterised by a necrotizing granulomatous inflammation with vasculitis. It belongs to the group of antineutrophil cytoplasmic antibodies- (ANCA-) associated vasculitis which are responsible for the inflammation of WG. The diagnosis of WG is typically based on the recognition of the clinical picture and the detection of ANCA in the serum, especially cANCA anti-PR3 [1, 2].

The sinonasal tract is the most frequently affected site within the head and neck, and this site may be the only affected organ by WG [3]. However, the diagnosis of WG may be a challenge either when the patient refers to an oncologically oriented department or when facing with image studies (i.e., CT scan and PET) suggesting a malignant disease. These eventualities may contribute to the underdiagnosis WG, thus leading to a delay in the correct therapeutic approach. 

As far as histology is concerned, the presence of necrotizing granulomas inflammation and vasculitis is the hallmark of WG. However, these pathognomonic features may be missed by the pathologists either in the early stage of the disease or when facing with small fragments of tissue. 

We herein present a case of sinonasal WG clinically and radiologically mistaken for an undifferentiated carcinoma of the nasopharynx.

## 2. Case Presentation

**Figure 1:**
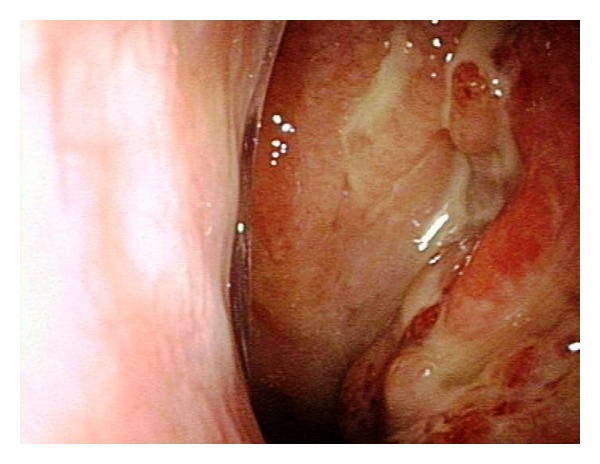
Fibroscopic exam showed the presence of an ulcerated mass of the rhinopharynx.

A 21-year-old girl was admitted to our hospital because of a 3-month history of otitis. A clinical evaluation, which included a nasal fibroscopic exam, revealed the presence of a vegetative and ulcerated mass of the rhinopharynx involving the left Eustachian tube, suggestive of malignancy (Figure 1).

**Figure 2:**
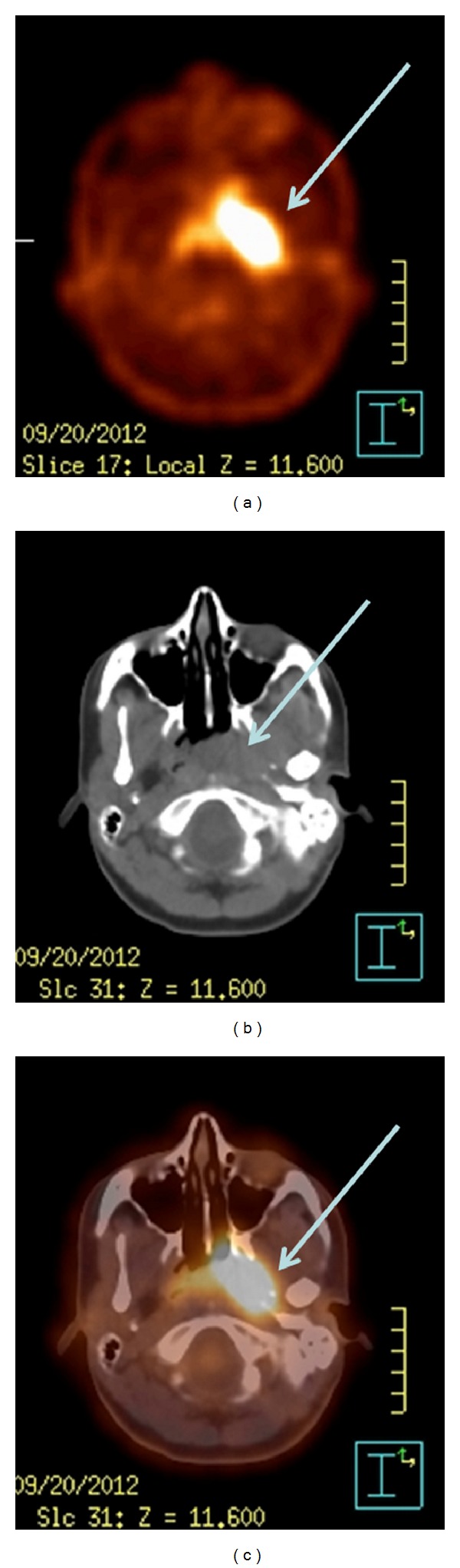
Images showing intense 18F-FDG uptake in rhinopharynx: (a) PET, (b) CT, and (c) PET-CT.

The presence of enlarged laterocervical nodes supported the clinical suspicious of malignancy. Her initial CT scan showed a large mass at left nasopharyngeal wall with features suggestive of neoplasia with local extension into the left parapharyngeal space laterally. For further evaluation, she had an FDG PET/CT scan (Gemini GXL; Philips, Holland). The whole body scan was performed 50 min after intravenous injection of 208 MBq (5.6 mCi) of 18F-FDG revealing a focal area of FDG-6-phosphate accumulation in rhinopharinx involving left parapharyngeal space (maximum standard uptake value: [SUV_max⁡_] = 13.2) (Figure 2).

Even the PET finding supported the clinical diagnosis of malignancy. A biopsy was performed. Histology was negative for carcinoma and lymphoma on the basis of morphological and immunohistochemical findings (normal distribution of cytokeratins: CD20, CD3, bcl-2, bcl-6, CD10, and CD5).

**Figure 3:**
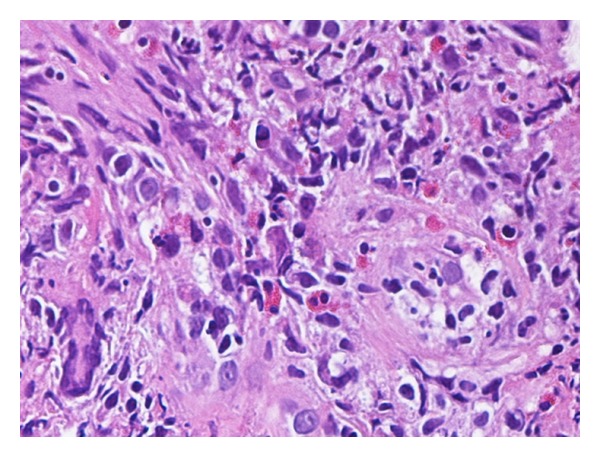
Histology revealed the presence of granulomatous inflammation with scattered giant cells and vasculitis.

Because of the clinical and radiological suspicions of malignancy, an additional biopsy was performed. Histological examination revealed morphological immunohistochemical findings identical to the previous ones leading to a descriptive diagnosis of “no evidence of malignancy.” An aspecific medical therapy was started. Since the clinical symptoms were not responsive to the therapy, a third biopsy was performed showing the presence of a granulomatous inflammation with scattered giant cells and vasculitis suggestive of Wegener's disease (Figure 3).

On the basis of these histological findings, a serum determination of ANCA was performed, showing strong (+ + +) cANCA positivity and thus supporting the histological diagnosis of WG. Clinical evaluation ruled out the pulmonary and renal involvement of the disease. The patients started atherapy with Rituximab (chimeric anti-CD20 monoclonal antibody) and suddenly improved the clinical symptoms.

## 3. Discussion

WG is an autoimmune disease histologically characterised by a necrotizing granulomatous inflammation associated with vasculitis of small and medium vessels. WG belongs to the group of antineutrophil cytoplasmic antibodies- (ANCA-) associated vasculitis which are responsible for the inflammation of WG. The determination of serum ANCA can assist in the diagnosis of WG, but ANCA positivity is not always conclusive for diagnosis, and the negativity for ANCAs is not sufficient to reject the diagnosis [1, 2].

Although lungs and kidneys are typically involved in the course of WG, many other organs, including the upper respiratory tract, may be affected [4, 5]. However, the typical necrotizing granulomas and vasculitis may be missed by the pathologists either in the early stage of the disease or in small fragments of tissue. Consequently, the diagnosis may be a challenge when the typical histological features are absent or when lungs and kidneys are not involved in disease or even the patient refers to an oncologically oriented department. Moreover, the radiological findings (i.e., CT scan and PET) may lead to an erroneous diagnosis because of the overlap features with other diseases including malignancies. Thus, unusual clinical presentation together with nonspecific radiological and histological features may delay the correct diagnosis leading to an erroneous therapeutic approach with dramatic clinical consequences [6].

In the present case, the suspicions of malignancy was highly supported either by the clinical presentation or by image analysis (namely, CT scan and PET/CT). Carcinomas of the sinonasal tract are highly aggressive neoplasm arising from the nasal cavity and paranasal sinus. Although sinonasal carcinoma is rare, it must be suspected when facing with an ulcerated mucosa of this tract even in young patients [7]. Clinical presentation is usually that of epistaxis, nasal obstruction, nasal discharge with occasional bloody drainage, and serious otitis media which may be among the earliest symptoms. Other symptoms include difficulty in breathing, cervical lymphadenopathy, and cranial nerve involvement [8]. The presentation of neck mass by the patients in the form of cervical lymphadenopathy reinforces the suspicion of metastatic carcinoma. Taking all these considerations into account, the clinical suspicious of carcinoma was the preferred clinical diagnosis in our case. 

Moreover, even the image analysis together with the PET study oriented and supported the clinical diagnosis of malignancy. Preliminary studies have reported promising results when malignant diseases in the head and neck were assessed with combined PET/CT since 18F-FDG PET has been reported sensitive for the diagnosis and staging of several types of malignancy [9, 10]. However, 18F-FDG accumulation is not specific to tumours, and 18F-FDG uptake in benign processes has been widely reported [11]. However, distinguishing malignant lesions from benign inflammatory or infectious processes is challenging because both can cause increased 18F-FDG uptake. SUVs have been proposed as being useful for discrimination. The cutoff value is controversial; although 2.5–3.9 is generally accepted, considerable overlap still exists between malignant and benign lesions [12, 13].

Although clinically suggestive of malignancy, a definite diagnosis relies on the histological confirmation of the disease. In the present case, two consecutive biopsies were negative for malignancy. It should be underlined that a clinical diagnosis different from “malignancy” should be suspected when facing with consecutive histological diagnosis of “no evidence of disease” on bioptic samples. WG should be one of the differential diagnoses of 18F-FDG PET/CT positive scans in nonmalignant conditions, in conjunction with appropriate clinical background. False positive results are possible with 18FDG-PET alone because an infectious or inflammatory process producing uptake might be present, and because physiologic uptake into structures such as tonsils, salivary glands, and muscles might occur [14, 15]. 

The recognition of WG in its early phase is of paramount importance since that if left untreated the disease runs an accelerated clinical course. Our case underlines that sinonasal involvement of WG may clinically overlap sinonasal carcinoma and should be included in the list of differential diagnoses of the masses of this region. On the other hand, pathologists have to consider that WG may not be histologically evident in small bioptic samples and that minimal changes including focal necrosis or granulomatous inflammation could be suggestive of WG. 

In conclusion, including the WG in the list of the differential diagnoses of sinonasal masses may have as a practical consequence to test serum ANCA in order to confirm or support the diagnosis of WG. This could have a more cost effectiveness effect in the diagnosis and treatment of an unusual but underdiagnosed condition of the sinonasal tract.
